# Measurement of Protein Synthesis Rate in Rat by [^11^C]Leucine PET Imaging: Application to the TgF344-AD Model of Alzheimer’s Disease

**DOI:** 10.1007/s11307-022-01796-0

**Published:** 2022-12-20

**Authors:** D. Bochicchio, L. Christie, C. B. Lawrence, K. Herholz, C. A. Parker, R. Hinz, H. Boutin

**Affiliations:** 1 Division of Neuroscience and Experimental Psychology, Faculty of Biology, Medicine and Health, School of Biological Sciences, University of Manchester, Manchester, M13 9PL UK; 2 Wolfson Molecular Imaging Centre, University of Manchester, Manchester, M20 3LJ UK; 3 Geoffrey Jefferson Brain Research Centre, Manchester Academic Health Science Centre, Northern Care Alliance & University of Manchester, Manchester, UK; 4 GlaxoSmithKline, Gunnels Wood Road, Stevenage, SG1 2NY UK; 5 Division of Informatics, Imaging and Data Sciences, Faculty of Biology, Medicine and Health, School of Health Sciences, University of Manchester, Manchester, M13 9PL UK

**Keywords:** Alzheimer’s Disease, TgF344-AD Rats, Amino Acid PET, Protein Synthesis rate, [^11^C]Leucine

## Abstract

Long-term memory requires stable protein synthesis and is altered in Alzheimer’s disease (AD). This study aimed to implement a method to measure the cerebral protein synthesis rate (PSR) with [^11^C]leucine PET *in vivo* in rats and evaluate potential PSR alterations longitudinally (6, 12 and 18 months old) in the TgF344-AD rat model of AD. Wistar, wild-type (WT) and TgF344-AD rats (TG) were scanned for 60 min with [^11^C]leucine. Arterial blood activity was monitored online and with discrete whole blood and plasma samples by γ-counting in Wistar rats, WT (*n* = 4) and TG (*n* = 5). Unlabelled amino acids were measured in plasma. The sensitivity of [^11^C]leucine PET to measure alterations in PSR was assessed in Wistar rats by injection of PSR inhibitor anisomycin before PET acquisition. Anisomycin administration significantly reduced the net uptake rate constant (*K*_cplx_) of [^11^C]leucine and PSR, proving the suitability of the method. For the longitudinal study, averaged population-based input functions were used to calculate PSR. We found a significant genotype effect on PSR (decrease in TG vs WT) only in the globus pallidus. This study suggests that [^11^C]leucine PET is sensitive enough to measure brain PSR in rat but that cross-sectional design with individual input function should be preferred.

**Supplementary Information:**

The online version contains supplementary material available at 10.1007/s11307-022-01796-0.

## Introduction

Alzheimer’s disease (AD) is a neurodegenerative disease and is the most prevalent form of dementia, affecting two thirds of the 50 million people suffering from dementia worldwide [1]. The main symptoms of AD include memory loss and confusion, while the pathological hallmarks are β-amyloid plaques, neurofibrillary tangles (NFT), neuronal and synaptic loss and chronic neuroinflammation [1] with oxidative stress, endoplasmic reticulum (ER) and mitochondrial dysfunction also described in AD [2]. These promote the activation of the unfolded protein response (UPR) pathway with the aim to restore cellular homeostasis. Activation of the UPR pathway, which has been found in post-mortem brains of AD patients and in animal models of AD [3–5], causes an attenuation of protein synthesis, which, if prolonged, causes cellular dysfunction and death [6]. This is of particular importance as it has been hypothesised that memory consolidation, which is impaired in AD, is due to *de novo* synthesis of proteins [7]. Therefore, the UPR pathway could represent a potential therapeutic target in AD. However, little is still known about alterations in protein synthesis rate (PSR) in AD and whether it can be measured by *in vivo* PET imaging. To determine PSR in the central nervous system (CNS) *in vivo,* [^11^C]leucine PET is preferred over other amino acids, such as [^11^C]methionine, as those have more complicated metabolic pathways which consequently complexify modelling and quantification [8, 9]. Leucine is transported across the blood–brain barrier through the large amino-acids transporter (LAT1) system. The LAT system is sodium-independent and binds amino acids with large neutral side chain (LNAA) including leucine, phenylalanine, tyrosine, isoleucine, methionine, valine, histidine and tryptophan; therefore, it is important to measure the concentration in LNAA as any change in concentration of one LNAA in plasma will affect the transport of the others [10]. Carboxyl-labelled leucine (referred as [^11^C]leucine from now on) is either incorporated in proteins or metabolised, ultimately leading to the production of CO_2_ and CO_2_ by-products [8]. The metabolism of leucine in brain was described by Schmidt et al. [8] who introduced the λ parameter to account for the contribution of proteolysis and [^11^C]leucine recycling during *de novo* protein synthesis (1-λ). Here, we have used the compartmental model from Schmidt et al. [8] simplified by Sundaram et al. [11], where there are no distinction between intra- and extra-cellular space and where ^11^CO_2_ fixation and ^11^CO_2_ in brain are considered negligible since they are low and at equilibrium with blood.

The aims of the present study were therefore to *(i)* implement the method to measure PSR in rats using [^11^C]leucine and *(ii)* apply this method to investigate potential changes in PSR in the TgF344-AD rat model of AD [12, 13] at 6, 12 and 18 months of age. TG rats are characterised by increased levels of Aβ plaque deposition and p-Tau, NTF, neuroinflammation, increased blood-brain barrier permeability, decreased functional connectivity and some cognitive dysfunctions, all aggravating with age [12–15]. Measuring PSR with [^11^C]leucine is technically demanding, so we initially developed our methodological approach in Wistar rats. In addition, to aid analysis, a population-based measurement of the input function and unlabelled amino acid measurement coming from the Fischer-344 strain were generated and used.

## Materials and Methods

### Animals

Male Wistar rats were purchased from Charles River (Margate, Kent, UK). Fourteen rats were scanned with [^11^C]leucine; all animals had online and discrete blood sampling; 6 were used for baseline scan, and 3 received a pre-injection of anisomycin (PSR inhibition) prior to PET scan, and an additional 5 rats were used for measurements of [^11^C]leucine and LNAA concentrations in blood and plasma on the bench only (i.e. not scanned). Two male and two female Fischer-344 (WT) and TgF344-AD (TG) rats with the APP_swe_ and PS1_Δe9_ mutations (purchased from the laboratory of Prof T. Town, University of Southern California) were set up as breeding pairs, housed in the Biological Services Unit at the University of Manchester for breeding purposes. Genotyping was outsourced to Transnetyx® (Cordova, USA). Male WT and TG rats were imaged via [^11^C]leucine PET at 6, 12 and 18 months of age (for details of *n* number, body weight and inclusion/exclusion, see Supplementary Tables 1 and 2). All procedures were conducted in accordance with the Animals (Scientific Procedures) Act 1986 and the GSK Policy on the Care, Welfare and Treatment of Animals. For the whole duration of the experiment, all animals were housed in groups of 2-4 per cage with individual ventilation, environmental enrichment, constant access to food and water and a 12:12-h light/dark cycle (7 AM to 7 PM). Although using other PET tracers, sample sizes were based on our experience using this [13] and other models of AD [16] and PET imaging. Investigators were not blind to the genotype of the animals. Exclusion criteria were related to potential health issues (spontaneous tumour, *etc*.) preventing scanning of the animals or leading to the animal to be culled to prevent unnecessary suffering. Whenever possible, animals that had died or were excluded before the 18 months time point were replaced by age-matched rats of the same genotype (for more details see Supplementary Tables 1 and 2).

### Scanning Protocol and Image Analysis

L-Leucine was labelled at the carboxyl acid group 1 with ^11^C as previously described [17] with a purity > 98% and a 37.5 ± 7.5 GBq/µmol molar activity. Rats were anaesthetised by isoflurane inhalation (induction 4–5% and 2–2.5% thereafter) in O_2_/NO_2_ (30%/70%), monitored for respiration and temperature using a pressure-sensitive pad and a rectal probe (BioVet, m2m Imaging Corp., USA) for the duration of the scans. Imaging was carried out on a Siemens Inveon® PET/CT scanner (for full details of acquisition and reconstruction protocols, see Supplementary materials). For all scans, 300 μl of tracer (injected dose 37.7 ± 7.45 MBq, molar activity 22.7 ± 18.7 GBq/µmol at injection time) and 300 μl of saline were injected in the tail vein sequentially using injection pumps (syringe pump, Cole-Palmer®, ref. WZ-74905–02) at a rate of 1.2 ml/min in a single bolus over 30 s at the start of a 60-min PET acquisition. To scale the population-based input function (PBIF) to each individual in the longitudinal study, images were segmented automatically using local means analysis (LMA) in BrainVisa 4.1.1 (http://brainvisa.info) [18–21], and the segmented region of interest (ROI) of the heart left ventricle was then selected. Skeletal and whole-brain ROIs were defined manually in the CT images using Anatomist 4.1.1 to register PET-CT images with the rat MRI template adapted from Schwarz and colleagues [22] used thereafter for quantification of atlas-based brain ROIs.

In order to assess the sensitivity of the [^11^C]leucine PET to measure PSR alterations, three Wistar rats were injected i.v. with 60 mg/kg of anisomycin in saline (pH 7) [23] 10 min prior PET acquisition. Anisomycin inhibits the protein synthesis by interfering with the 80S ribosome, and the effect is rapid (within 15 min) and reversible (lasts 90 min), 60 mg/kg producing an 80-96% inhibition of the PSR [23, 24]. Images are shown as standard uptake values (uptake in Bq.cm^−3^ × 10^−6^ × body weight (g)/injected dose in MBq, hence normalising uptake for injected dose and body weight).

### Input Functions

Individual arterial input functions (AIF) were determined for each Wistar rat (*n* = 6 baseline and *n* = 3 with anisomycin) and WT and TG rats at 12 (*n* = 3) and 18 months (*n* = 6) (due to unexpected loss of animals and other experimental constraints, the *n* number were low for blood data in WT and TG, and both genotypes were pooled together) (see details in Supplementary Table 3). A femoral arteriovenous shunt was connected to a Swisstrace™ Twilite® system for continuous monitoring of whole blood radioactivity (full details in Supplementary materials). Whole blood samples were collected from the shunt at 2, 5, 10, 20, 30, 40 and 60 min post-injection into heparinised Eppendorf tubes and placed immediately on ice. Aliquots of whole blood and plasma were counted using a γ-counter, and additional plasma aliquots were stored at -80 °C (full details in Supplementary materials) until sent to Alta Bioscience Ltd (Redditch, UK, https://altabioscience.com) for analysis of the large neutral amino acids (LNAA) (histidine, methionine, leucine, isoleucine, valine, phenylalanine, tyrosine and tryptophan), concentration in nmol/ml [25]. The plasma/whole blood and free/protein-incorporated [^11^C]leucine ratios were measured by γ-counting (full details in Supplementary materials). For the longitudinal study, the PSR was calculated using population-based values of unlabelled leucine, a population-based AIF scaled for each rat using an image-derived input function derived from quantification of the activity in the left ventricle (full details in Supplementary materials and Figs. S1 and S2) and the average plasma/whole blood ratio and free/protein-incorporated [^11^C]leucine in plasma ratio obtained in the F344 rats (Fig. 3D).

### Modelling

**Fig. 1:**
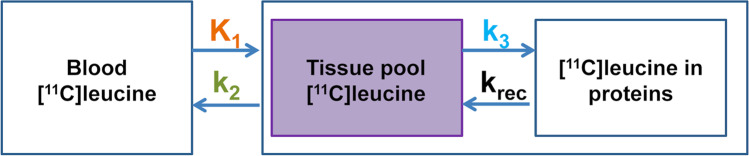
Simplified compartmental model of leucine metabolism in the brain. The rate constants *K*_1_ and *k*_2_ represent the transfer of leucine across the blood-brain barrier from blood to the brain and *vice versa*. *k*_3_ represents the incorporation of leucine in proteins, and *k*_rec_ (recycling of leucine from the protein pool) is negligible (adapted from[11]).

In-house software MICK (Modelling, Input functions and Compartmental Kinetics), written in MATLAB, was used to determine the PSR through the calculation of the three rate constants (Fig. 1), *K*_*cplx*_ and λ [11]. The compartmental model used to study the [^11^C]leucine brain uptake is shown in Fig. 1.

λ is defined as

1 $$\underset{t\to \infty }{\mathrm{lim}}[\frac{\frac{Cf}{Cf\left(cold\right)}}{\frac{Cp}{Cp\left(cold\right)}}]$$

where *Cf* is the concentration of free leucine in the precursor pool in the tissue and *Cp* is the concentrations of leucine in plasma (cold = unlabelled leucine). Unlabelled leucine is at steady state in tissue and:

2 $$\frac{{dC}_{f(cold)}}{{d}_{t}}=\frac{{dC}_{p(cold)}}{{d}_{t}} = 0$$

and because *Cf* for labelled leucine is equal to

3 $${C}_{f}=\frac{K_{1}\times C_{p}}{(k_{2} + k_{3})}$$

λ will be [11]

4 $$\lambda =\frac{{k}_{2}}{{k}_{2}+{k}_{3}}$$

The unidirectional uptake rate of plasma leucine into tissue *K*_*cplx*_ is calculated as follows [11]:

5 $${K}_{cplx}=\frac{{K}_{1}{k}_{3}}{{(k}_{2}+{k}_{3})}$$

For [^11^C]leucine, it is assumed that none is recycled from radioactive proteins during the experimental time of 60 min; *k*_rec_ = 0. Therefore, the PSR can be estimated by the following equation:

6 $$PSR={K}_{cplx}\times \frac{leucine [C]}{\lambda }$$

where *PSR* is in μM·min^−1^, *K*_*cplx*_ in min^−1^ and *leucine[C]* is the concentration of unlabelled leucine in arterial plasma in nmole·ml^−1^.

### Statistical Analysis

GraphPad Prism 9.4 was used to analyse the data. All data are expressed as mean ± SD. Effects of anisomycin on *K*_*cplx*_ and PSR were analysed using a two-way ANOVA (treatment and ROIs as main factors and interaction treatment × ROIs). Two-way ANOVA (age and origin of samples (arterial vs venous) as main factors and interaction age × origin) and Šídák *post hoc* tests were used to compare the differences in concentration of unlabelled leucine between arterial and venous plasma samples in WT rats at 12 and 18 months. PSR and SUV in the longitudinal study were analysed using mixed effect analysis (age and genotype as main factors and interaction age × genotype) and Šídák *post hoc* tests.

## Results

### Administration of Anisomycin Induced a Significant Inhibition of Cerebral Protein Synthesis That Is Measurable by [^11^C]Leucine PET

**Fig. 2:**
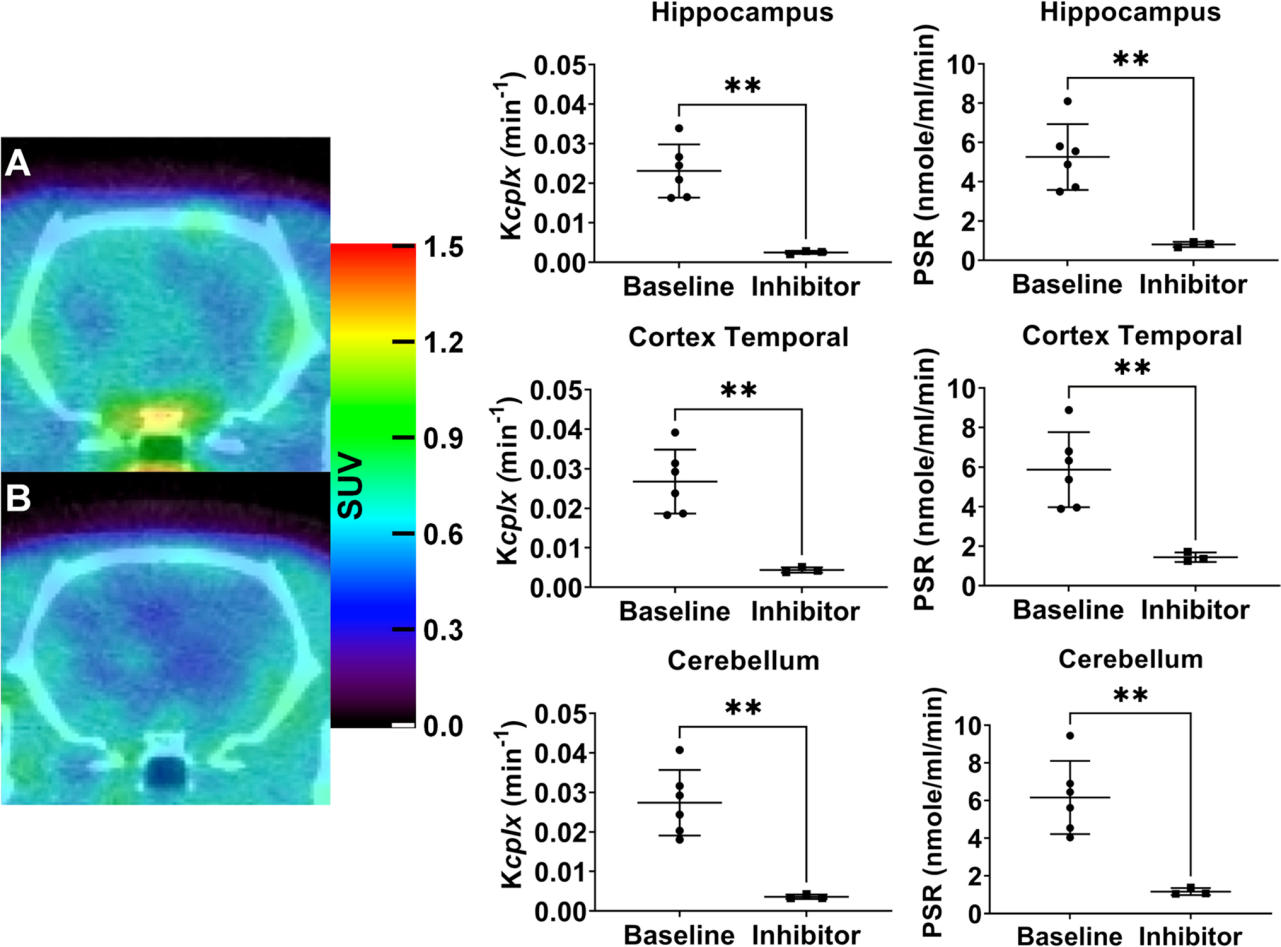
Representative sum 20-60 min PET images of [^11^C]leucine uptake (shown as SUV) in the brain without (**A**) and with (**B**) injection of anisomycin (inhibitor) at 60 mg/kg i.v. The graphs show the comparison of *K*_cplx_ (left column) and PSR (right column) at baseline and after anisomycin injection. Data analysed with two-way ANOVA (treatment and ROIs as main factors and interaction treatment × ROIs) and Šídák *post hoc* tests. **p ≤ 0.01. Data were expressed as mean ± SD.

In order to determine whether [^11^C]leucine PET is sensitive enough to detect inhibition of the PSR, we used the potent protein synthesis inhibitor [23] anisomycin, which inhibits the 80S ribosome system, in Wistar rats. Injection of anisomycin (60 mg/kg i.v.) did not lead to any detectable physiological effect as previously described [23]. A significant inhibition of *K*_*cplx*_ and PSR was observed following anisomycin pre-treatment across all ROIs studied (77–89%; Fig. 2) (time-activity curves and compartmental modelling fits are shown in Fig. S3) (e.g. in hippocampus, PSR_baseline_ = 5.25 ± 1.67 nmol/ml/min vs PSR_anisomycin_ = 0.81 ± 0.13 nmol/ml/min, *p* = 0.005).

### Analysis of Unlabelled Amino Acids in Blood Samples

To assert *(i)* potential differences between strains of rats and *(ii)* the usability of venous instead of arterial blood sampling, we compared arterial and venous samples from Wistar and F344 rats.

**Fig. 3:**
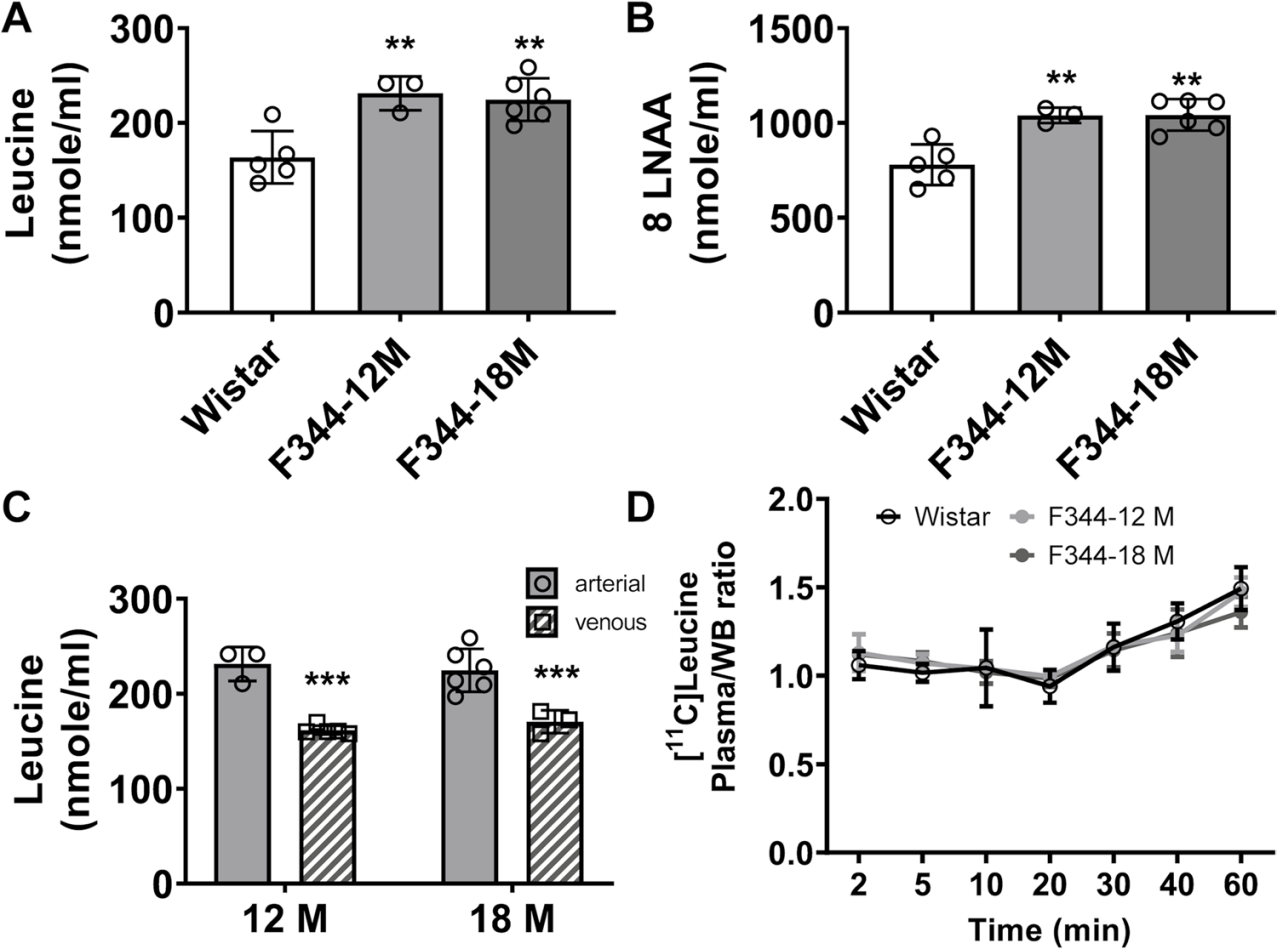
Significant difference in unlabelled leucine (**A**) and 8 large neutral amino acids (LNAA) (**B**) concentrations in arterial plasma samples from Wistar and F344 rats at 12 and 18 months. (**C**) Significant differences in unlabelled leucine between arterial and venous samples in F344 rats at 12 and 18 months. (**D**) No significant differences in plasma/whole blood (WB) ratio between Wistar and F344 rats at 12 and 18 months. Data analysed by one- (**A** and **B**) or two-way ANOVA (**C** and **D**) and Šídák *post hoc* tests. **p ≤ 0.01; ***p ≤ 0.001. Data expressed as mean ± SD.

F344 rats had significantly higher concentrations of unlabelled leucine (+ 41% and + 37% at 12 and 18 months, respectively) than Wistar rats (Fig. 3A). Moreover, the concentrations of the LNAA were significantly higher in the F344 than in the Wistar strain (+ 33%) (Fig. 3B). In addition, there were significant differences in unlabelled leucine concentrations between arterial and venous blood: 231 ± 17.9 nmol/ml vs 162 ± 4.7 nmol/ml (12 months, -30%, *p* = 0.0001) and 225 ± 22.5 nmol/ml vs 171 ± 11.9 nmol/ml (18 months, -24%, *p* = 0.001) in F344 rats (Fig. 3C). Conversely, there was no significant difference in plasma/whole blood ratio between Wistar and F344 rats, and this measure was robustly consistent between F344 rats (Fig. 3D). Resulting individual *K*_cplx_, PSR and λ values in Wistar and F344 rats are presented in Supplementary Table 3. Consequently, the group average plasma/whole blood ratio in F344 rats was used in the modelling of the longitudinal studies.

### Validation of a Population-Based Input Function in Fischer-344 Rats

**Fig. 4:**
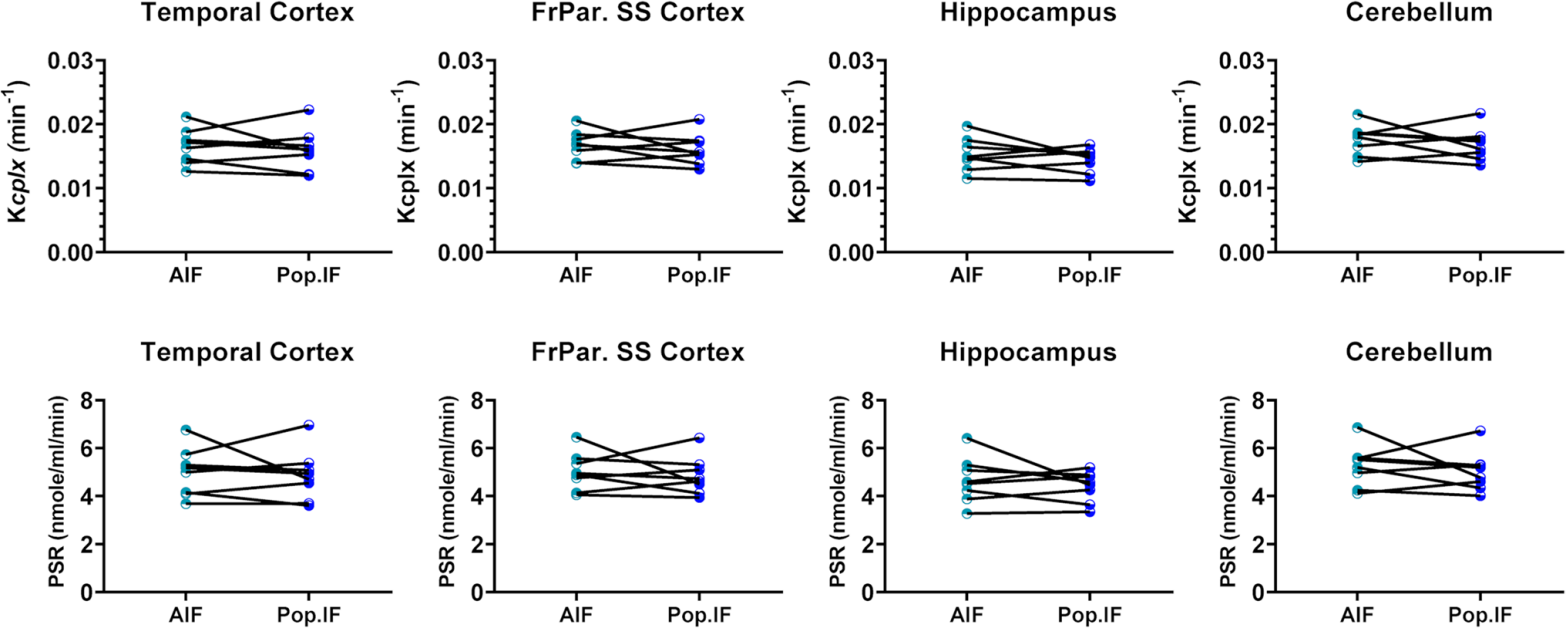
Comparisons of PSR values determined with individual arterial input function and population-based input function in various brain regions of 12 and 18 months old WT and TG rats. Data were analysed using 2-way ANOVA; no significant difference was found. FrPar. SS, frontoparietal somatosensory cortex.

Subsequently, for the longitudinal study, a PBIF (Figs. S1 and S2) was validated by comparing the PSR obtained with this PBIF against the PSR calculated from individual AIF. Our results showed no significant difference between the PSR calculated with the two IF (Fig. 4) supporting the use of such PBIF for the longitudinal study.

### Measurement of PSR in WT and TG Rats

**Fig. 5:**
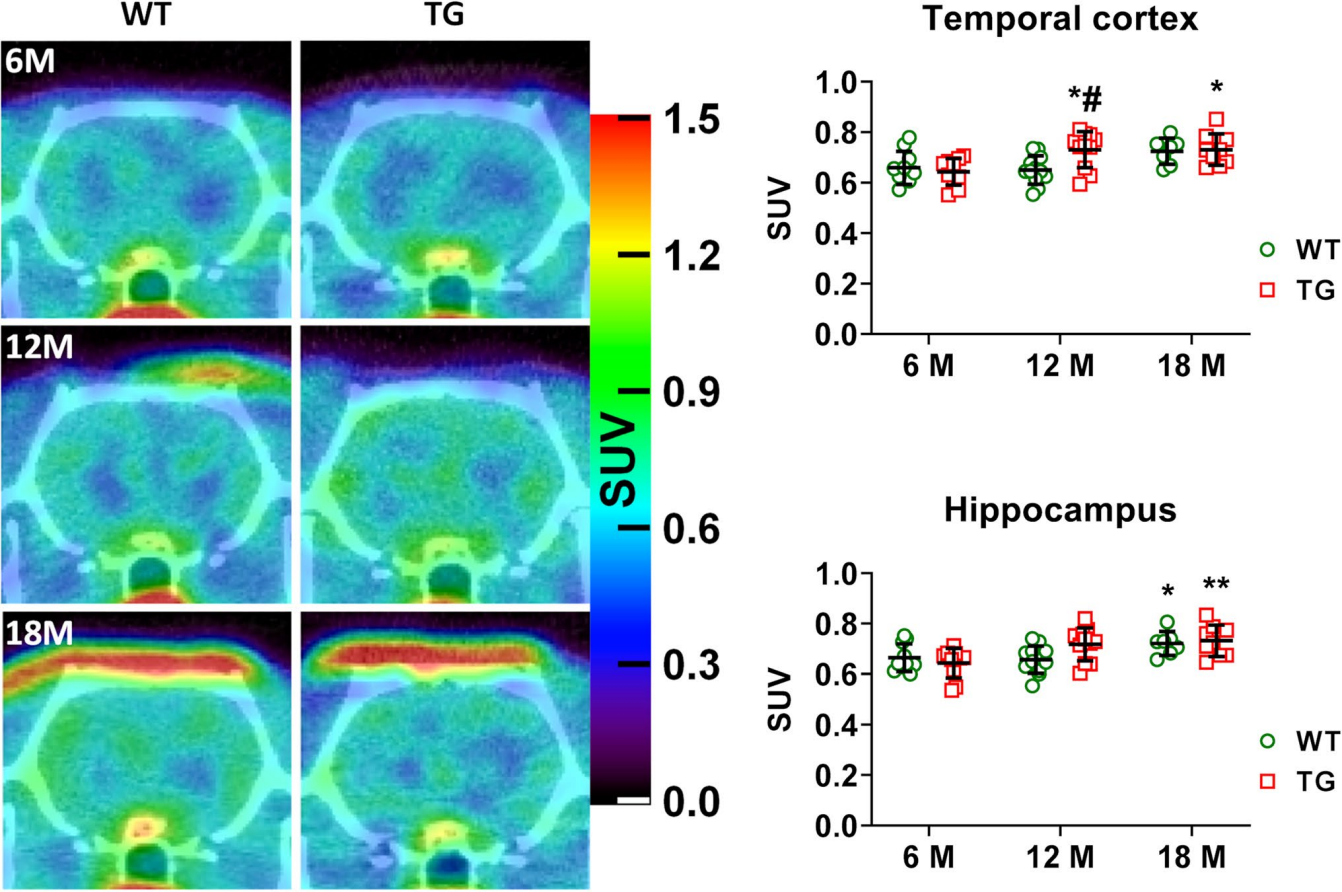
Representative summed 20-60 min PET images of [^11^C]leucine uptake, shown as SUV, in the brain of a WT and TG rats at 6, 12 and 18 months. Data analysed using mixed model effect and Šídák *post hoc* test. Data expressed as mean ± SD. * or ** indicates significant differences with the 6 months’ time-point, and # indicates significant difference vs age-matched WT. # or * *p* ≤ 0.05; **p ≤ 0.01.

[^11^C]Leucine uptake in WT and TG rats was first measured using standard uptake values (SUV) (Fig. 5). The SUV analysis in the hippocampus and temporal cortex showed a significant age effect *p* = 0.001 in both brain regions) and genotype × age interaction (*p* = 0.032 and *p* = 0.017, respectively) (Fig. 5). More specifically, SUV increased in both genotypes at 18 months in WT (+ 8%, *p* = 0.012) and TG (+ 11%, *p* = 0.006) vs 6 months in the hippocampus and in the temporal cortex of TG rats at 12 (+ 14%, *p* = 0.039) and 18 months (+ 14%, *p* = 0.003) vs 6 months. The *post hoc* tests showed a significant difference between WT and TG only in temporal cortex at 12 months (*p* = 0.024) (Fig. 5).

However, [^11^C]leucine has complex uptake and incorporation processes, and SUV quantification is not sufficient to accurately represent the PSR; therefore, we also performed kinetic modelling using a compartmental model as previously described by Sundaram et al. [11] to report PSR values in nmole/ml/min rather than just SUV in WT and TG rats.

This compartmental modelling showed no significant effect of genotype, age or interaction genotype × age on PSR values in all brain regions analysed (hippocampus, temporal cortex, cerebellum, cortex somatosensory, frontal cortex, thalamus, cortex cingulate/anterior and whole brain).

## Discussion

The aim of this project was first to implement the use of [^11^C]leucine PET to measure PSR and potential changes in PSR in rats. To study protein synthesis *in vivo*, different amino acids have been used [26–28]. For PET imaging, the most suitable amino acids to study protein synthesis are radiolabelled at the carboxyl group which results in low level of radiolabelled metabolites and hence provides an optimum approach for quantification [29]. Additionally, the ideal radiolabelled amino acid should also have a high brain uptake, only be incorporated into proteins and not be involved in other complex catabolic pathways [26]. [^11^C]Leucine fulfils these characteristics.

To validate the ability of [^11^C]leucine PET to measure changes in PSR *in vivo*, we administered the PSR inhibitor anisomycin at (60 mg/kg) 10 min prior to the PET acquisition. This dose induced a PSR inhibition of ~ 86% vs baseline in all brain regions analysed (Fig. 2). Our results are consistent with published data, where anisomycin injection induced a dose-dependent inhibition of ~ 80% at 60 mg/kg [23]. However, the modelling of these data required an arterial input function and blood sampling to measure unlabelled LNAA concentrations in plasma, an invasive and terminal method incompatible with longitudinal studies performed in expensive transgenic models of neurodegenerative diseases.

Confident that [^11^C]leucine PET was sensitive to detect changes in PSR, we went on to test a method without the need for arterial cannulation and blood sampling. We assessed potential differences between strains and arterial and venous samples to test the possible use of *(i)* a rat strain more readily available in the UK and less expensive such as Wistars and *(ii)* easier obtainable venous samples instead of arterial samples. Comparison of blood samples revealed significant differences in leucine and LNAA concentrations between Wistar and F344 strains and between arterial and venous samples. Differences in unlabelled leucine concentrations between species have been reported before, but to our knowledge, this is the first report showing differences between strains of a same species [11, 30–33]. Unfortunately, these findings compromised our intended use of a Wistar-based PBIF along with venous sampling for the longitudinal study in WT and TG rats. Our first analysis of the SUV showed modest but significant differences between genotypes and with age (Fig. 5). However, based on the complex uptake and incorporation of leucine in proteins, compartmental modelling is essential for an accurate measure of PSR. We therefore decided to build a PBIF in WT and TG (see Supplementary material and Figs. S1–S2) to calculate the PSR. Due to unexpected loss of animals and other experimental constraints, the number of WT and TG available to do this was unfortunately low, and we decided to pool the blood data by genotype rather than age, as previous reports have showed changes in PSR with ages [30]. Although blood data were very consistent across WT and TG and ages, we must acknowledge that this is a limitation in the present study. However, this study also proves that, for measuring parameters such as PSR which requires complex modelling, a cross-sectional study design should be preferred to a longitudinal study design. Indeed, and although more laborious, requiring more animals and hence more expensive, a cross-sectional study design allows individual arterial input function measurements which are not incompatible with a longitudinal design since femoral artery cannulation is a terminal procedure. To validate the use of this PBIF in the longitudinal study, we compared the PSR calculated with this PBIF against the PSR calculated with individual AIF (see Figs. S1 and S2) in a subset of F344 rats and observed no significant differences in PSR between the 2 methods (Fig. 4).

**Fig. 6:**
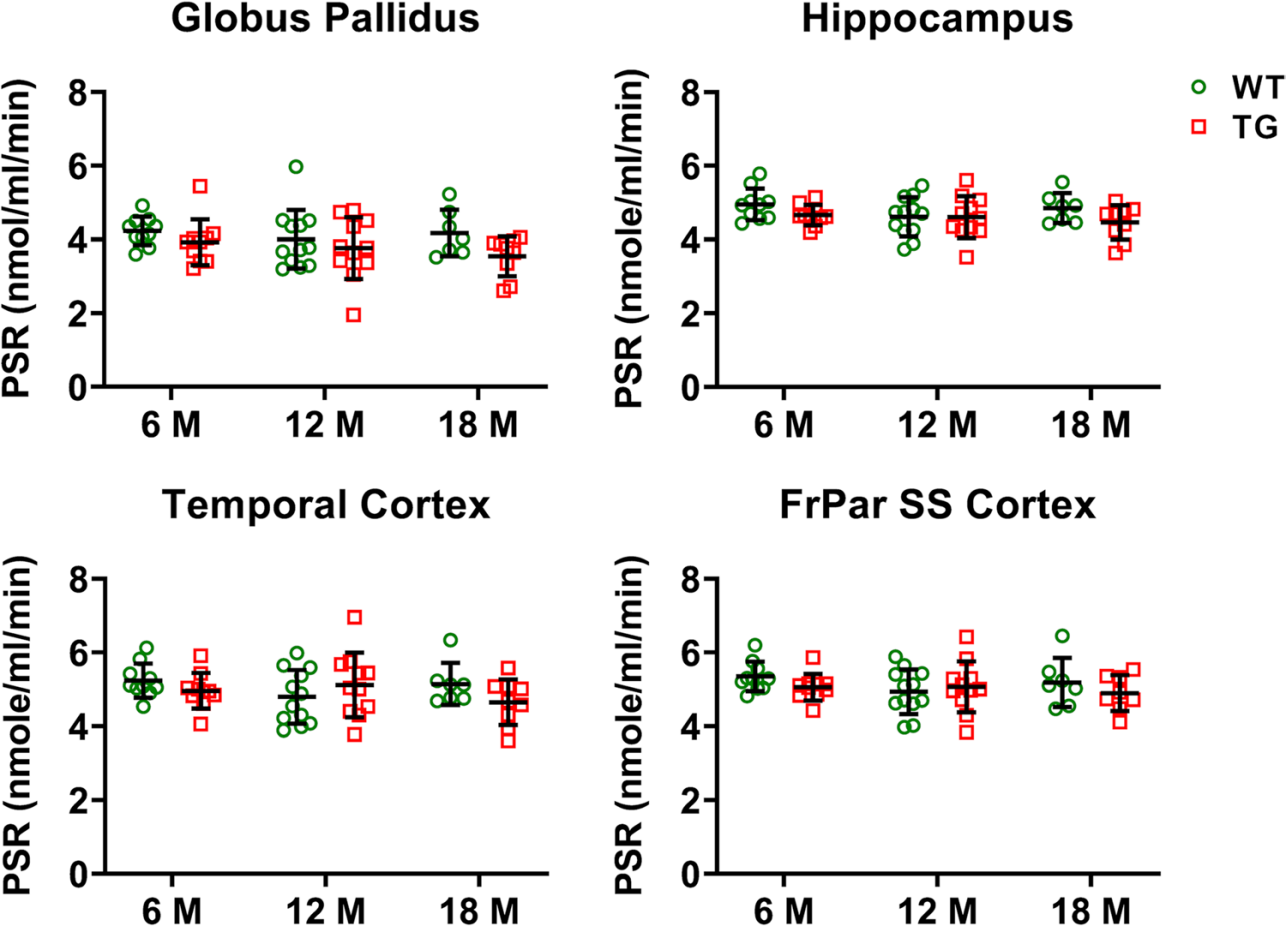
PSR in different brain regions of WT and TG rats at 6, 12 and 18 months. No significant effect of genotypes, ages or interaction genotype × age was detected. Data analysed by mixed model effect. Data expressed as mean ± SD.

Using this PBIF, our analysis showed no significant differences in PSR between age and genotype (Fig. 6). Despite the concentrations in leucine and LNAA in plasma, the plasma/whole blood and free/protein-incorporated ratio having low variability in F344 rats and that the comparison between *K*_cplx_ and PSR measured with AIF and PBIF showed no significant difference in the validation subgroup, the use of population-based average may have reduced group differences, potentially contributing to the lack of changes observed here. However, it must be noted that, here, the measures of *K*_cplx_ and PSR seem robust (low variability) (Fig. 6 and Supplementary Table 3).

In the brain as a whole region of interest, there are known PSR values in humans [32] and monkeys [9] which are reportedly lower (-62% and -44%, respectively) than those reported in this manuscript. Conversely, Sprague-Dawley rats have been reported to have higher PSR in all brain regions [30], but this previous study used a different methodology to ours and used very young (10-60 days post-natal, i.e. developing) animals. Sun et al. [30] showed that the incorporation of leucine in protein peaked at 10 days post-natal and decreased by 40-50% at post-natal day 60, supporting the hypothesis that during brain development and at a younger age, protein synthesis is at its peak and declines with age. This observation combined with data from this manuscript corresponds with that by Smith et al. [34] who also showed a gradual decline in PSR in Sprague-Dawley rats between 6 and 23 months of age. Overall, and although the methodology used by Smith et al. [34] was different, our PSR values are more comparable with theirs than those in very young animals [30], with similar lower values in the globus pallidus (~ 4 nmol/ml/min) and higher values in hippocampal and cortical areas. However, our cortical and hippocampal values were slightly lower than those obtained *ex vivo* by Smith et al. [34] (6 months WT vs 6 months Sprague-Dawley rats, frontoparietal somatosensory cortex, 5.35 ± 0.40 nmol/ml/min in vs 9.3 ± 0.3 nmol/g/min; hippocampus, 4.85 ± 0.41 nmol/ml/min vs 7.1 ± 0.3 to 14.8 ± 1.2 nmol/g/min in various part of the hippocampus) (see Table 5 of ref. [34]). The differences observed between these two studies might be due to differences in both the strain used and methodologies: i.e. *in vivo* [^11^C]leucine PET with fairly large ROIs and potential issues of partial volume effect vs *ex vivo* microdissection of small brain region and [^3^H]leucine quantification and modelling).

## Conclusion

Data obtained in this study confirm and support the use of [^11^C]leucine PET as a means of measuring PSR *in vivo* in rats. This first study in a model of neurodegenerative disease did not find any difference in brain PSR between WT and TG rats at any age in this model of AD. However, considering the limitations of using a PBIF discussed above, this study recommends the use of individual blood samples to establish the IF and measure the concentrations of unlabelled LNAA, hence favouring a more laborious and expensive cross-sectional study design over a longitudinal setting, to refine the measure of the PSR in future studies using AD or other models of neurodegenerative diseases.

## Supplementary Information

Below is the link to the electronic supplementary material.

Supplementary file1 (DOCX 35 KB)

Supplementary file2 (PDF 474 KB)
