# Supplementary material for: Measurement of Protein Synthesis Rate in Rat by [11C]Leucine PET Imaging: Application to the TgF344-AD Model of Alzheimer’s Disease
Source: Mol Imaging Biol. 2022 Dec 20;25(3):596–605. doi: 10.1007/s11307-022-01796-0 (PMC10172255; doi:10.1007/s11307-022-01796-0)
Supplement: Supplementary file 1 — Supplementary file1 (DOCX 35 KB) [file 11307_2022_1796_MOESM1_ESM.docx]

**Supplementary table 1:** Summary of the number of rats included or excluded in this study with the cause of exclusion.

|  |  | **Exclusion criteria applied** | | | |  |
| --- | --- | --- | --- | --- | --- | --- |
|  | **Total n number** | **SAH** | **Tumour/leukaemia** | **Unknown cause / other** | **Technical issues** | **Total included^✝^** |
| **Wistar** | 21 | 0 | 0 | 0 | 7***** | **14**** |
| **WT** | 14 | 1**^pm^** | 1**^#^** | 1**^##^** | 2**^###^** | **14** |
| **TG** | 19 | 1**^¶^** | 2**^¶¶^** | 3**^¶¶¶^** | 0 | **13** |

***^✝^*** *Included in at least one time-points for WT and TG.* ***SAH****: subarachnoid haemorrhage.* ******* *Four rats were used for attempts of measuring [^11^C]leucine and unlabelled leucine by HPLC in-house (unsuccessful, not reported in results), 3 other rats were excluded: 2 because low tracer purity (<98%) and 1 because of issues with the femoral canulation and arterial sampling.* ******** *Six rats for baseline measurements of PSR, 3 for inhibition with anisomycin, 5 for measurements of [^11^C]leucine concentration in blood and plasma on the bench only.* ***Pm*** *= excluded after post-mortem examination after the 18 months’ time-points scan.* ***^#^*** *Culled because of presumed leukaemia.* ***^##^*** *died after 12 months’ time-point scan.* ***^###^*** *one failed arterial canulation and 1 failed tracer production post-arterial canulation at 18 months’ time-point.* ***^pm, #, ##, ###^*** *All these WT were included in the 6- and 12-months’ time-points analysis.* ***^¶^*** *18 months old replacement rat culled because of SAH before scan.* ***^¶¶^*** *One culled at 8 months of age because of head tumour and one 18 months old replacement rat died of presumed leukaemia before scan.* ***^¶¶¶^*** *Culled because of overgrowing teeth at young age.*

**Supplementary table 2:** number of rats included in the study with body weight, injected dose and injected mass (mean±SD).

|  | **Baseline** | **Anisomycin** |  |
| --- | --- | --- | --- |
| **Wistar** | 11 (449±65g)  39.8±11.7MBq  6.6±4.6nmol | 3 (428±4.6g)  37.5±3.1MBq  12±6.1nmol |  |
|  | **6 months** | **12 months** | **18 months** |
| **WT** | 10 (420±19g)  33.1±5.1MBq  7.1±10.5nmol | 12 (473±18g)  38.3±11.4MBq  2.8±2.0nmol | 7 (481±31g)  41.2±2.9MBq  5.3±8.8nmol |
| **TG** | 9 (445±28g)  38.4±4.4MBq  3.4±4.3nmol | 11 (493±23g)  35.4±4.7MBq  2.0±1.2nmol | 9 (487±27g)  38.7±5.2MBq  1.4±0.9nmol |

**Supplementary table 3:** individual K_cplx_, PSR and λ values in the hippocampus of Wistar and 12 and 18 months old Fischer-344 rats measured with arterial input function.

| Wistar | | | |
| --- | --- | --- | --- |
| Rat ID | K_cplx_ (min^-1^) | PSR (nmole/ml/min) | λ |
| a02958 | 0.027 | 5.800 | 0.752 |
| a03256 | 0.016 | 3.497 | 0.770 |
| a03259 | 0.021 | 4.882 | 0.700 |
| a03270 | 0.016 | 3.721 | 0.715 |
| a03353 | 0.024 | 5.558 | 0.720 |
| a03355 | 0.034 | 8.100 | 0.685 |
| Mean ± SD | **0.023±0.007** | **5.260±1.677** | **0.724±0.032** |
| F344-12M | | | |
| Rat ID | **K_cplx_ (min^-1^)** | **PSR (nmole/ml/min)** | **λ** |
| a03147 (WT) | 0.014 | 4.515 | 0.725 |
| a03148 (TG) | 0.015 | 4.593 | 0.734 |
| a03149 (WT) | 0.013 | 3.879 | 0.752 |
| Mean ± SD | **0.014±0.001** | **4.33±0.39** | **0.737±0.014** |
| F344-18M | | | |
| Rat ID | **K_cplx_ (min^-1^)** | **PSR (nmole/ml/min)** | **λ** |
| a02907 (TG) | 0.015 | 4.230 | 0.788 |
| a02920 (TG) | 0.015 | 4.450 | 0.762 |
| a02927 (TG) | 0.016 | 5.072 | 0.732 |
| a02929 (WT) | 0.020 | 6.411 | 0.696 |
| a02930 (TG) | 0.011 | 3.268 | 0.797 |
| a02932 (WT) | 0.021 | 7.100 | 0.678 |
| Mean ± SD | **0.016±0.004** | **5.09±1.43** | **0.742±0.049** |
