# Supplementary material for: Measurement of Protein Synthesis Rate in Rat by [11C]Leucine PET Imaging: Application to the TgF344-AD Model of Alzheimer’s Disease
Source: Mol Imaging Biol. 2022 Dec 20;25(3):596–605. doi: 10.1007/s11307-022-01796-0 (PMC10172255; doi:10.1007/s11307-022-01796-0)
Supplement: Supplementary file 2 — Supplementary file2 (PDF 474 KB) [file 11307_2022_1796_MOESM2_ESM.pdf]

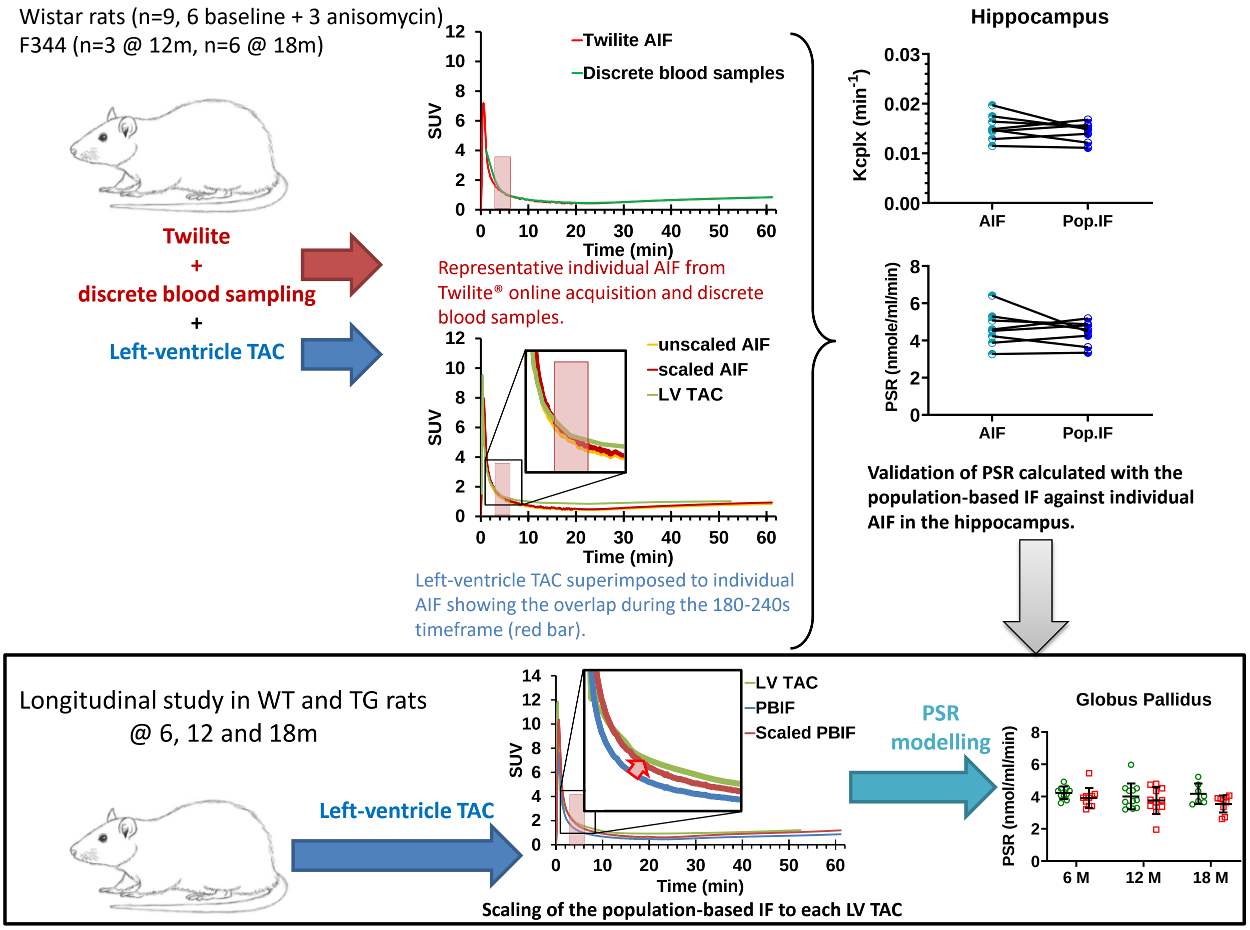

Figure S1: Schematic of the procedure followed to generate and validate the population-based input function and application of this population-based input function to the longitudinal study.

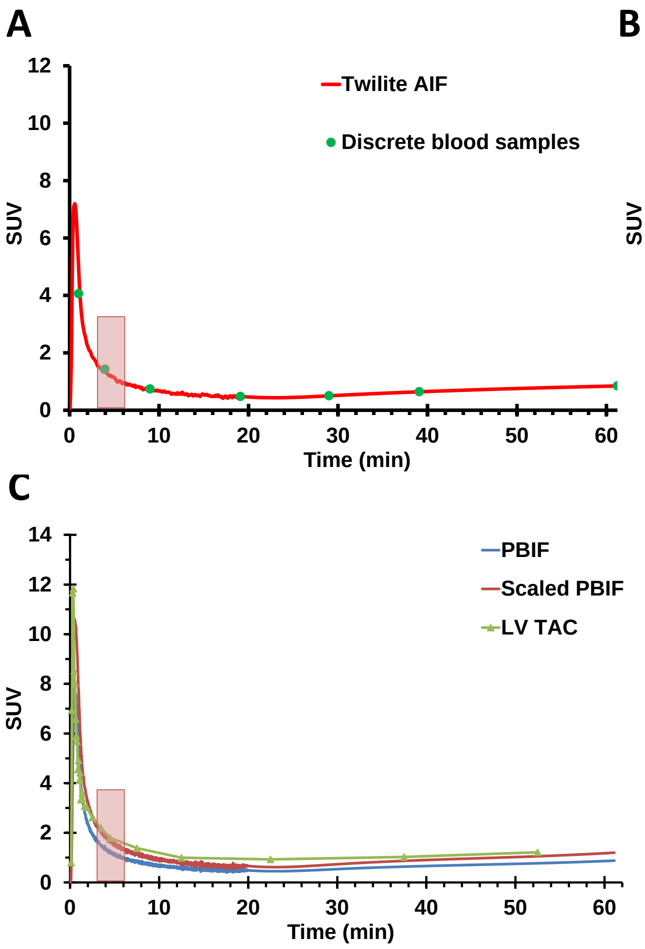

Figure S2: (**A**) Representative curves of arterial blood input function (AIF) as measured online by the Twilite® and discrete blood sampling, (**B**) representative example of an individual AIF and scaled AIF and Left Ventricle time-activity curve (LV TAC). Overlap (red bar) between the discrete blood samples, Twilite® AIF (**A**), unscaled and scaled AIF, and LV-TAC (**B**) was consistently observed between 180-240s, therefore this timeframe was chosen to calculate the ratio between LV TAC and Population-based IF (PBIF) to perform the scaling of the PBIF. (**C**) Representative example of the Population-based IF (PBIF) before and after scaling using the individual LV TAC.

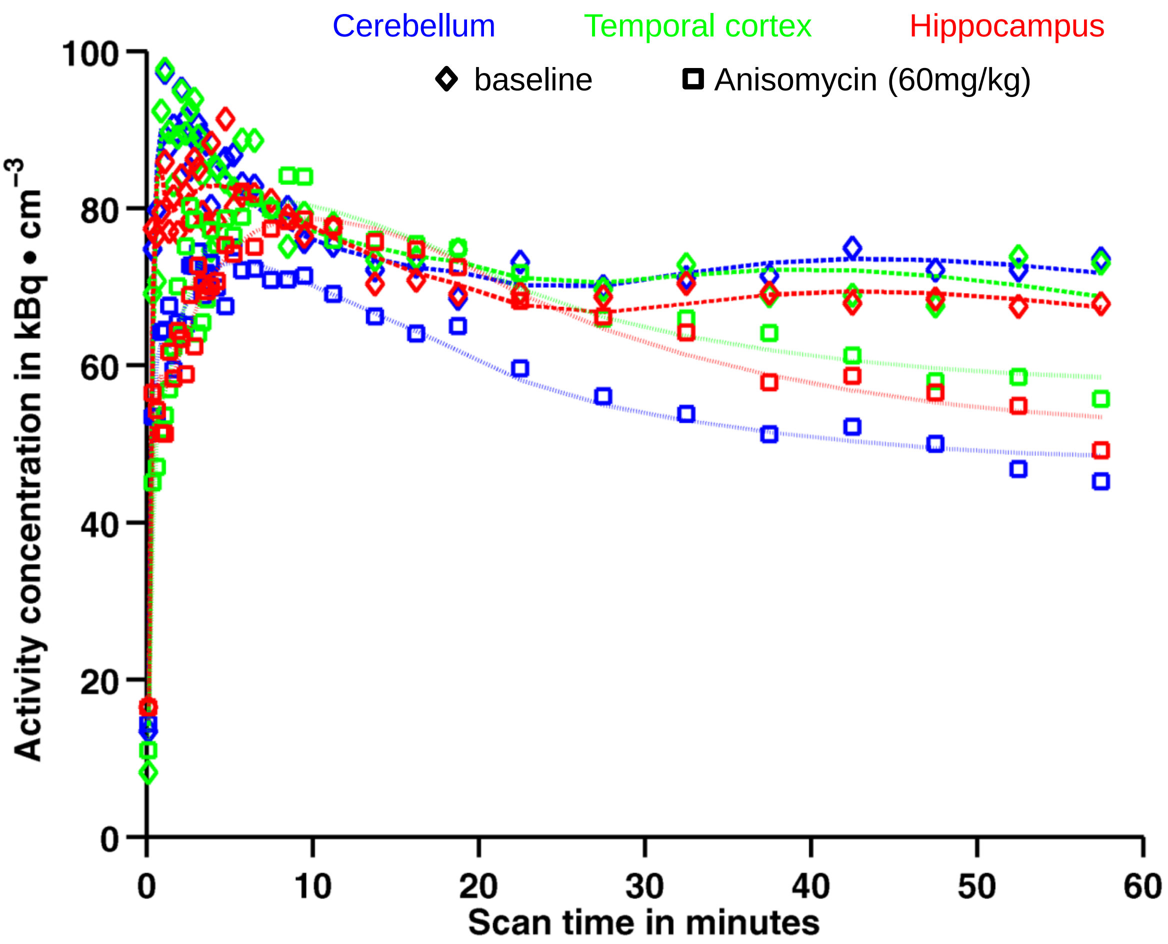

Figure S3: Time-activity curves of [^{11}C]leucine uptake in the cerebellum (blue), temporal cortex (green) and hippocampus (red) without (diamond symbols) and with anisomycin (square symbols) administered 60mg/kg 10 min prior to the injection of [^{11}C]leucine. The dashed lines represent the fits of the compartmental modelling to the measured tissue time-activity curves.

Supplementary

Scanning Protocol

A Siemens Inveon® PET/CT was used for this imaging study. A CT attenuation scan was performed before the 60min PET acquisition. The time coincidence window was set to 3.432 ns and levels of energy discrimination to 350 keV and 650 keV. List mode data from emission scans were histogrammed into 25 dynamic frames (14 × 5 s; 3 × 15 s; 3 × 1 min; 2 × 5 min; 3 × 15 min) for the quantification of the heart pool and into 36 dynamic frames (16 × 15 s; 4 × 30s; 4 × 1 min; 4 × 2.5 min; 8 × 5 min) for the analysis of the brain [^{11}C]leucine uptake. Emission sinograms were normalised, corrected for attenuation, scattering and radioactivity decay and reconstructed using OP-OSEM3D-MAP (16 subsets, 2-iterations of OP-OSEM3D followed by 18 iterations of MAP).

Arterial Input Function (AIF)

The femoral vein and artery were cannulated with a 26G catheter (Terumo® Surflo®-W) connected to a fine bore LDPE tubing (Smiths Medical™ Portex™, ref. 800/100/200, ThermoFisher Scientific UK, internal diameter 0.58 mm, external diameter 0.96 mm) to form an arterio-venous shunt running through a Twilite® Swisstrace™ coincidence counter (Swisstrace GmbH, Switzerland). Blood flow was maintained constant in the shunt by a peristaltic pump (Ismatec®) (350µl.min^{-1}). Whole blood samples (350-500 µl each) were collected immediately after the peristaltic pump through a T-connector at 2-, 5-, 10-, 20-, 30-, 40-, and 60-min post-injection into heparinised Eppendorf tubes and placed immediately on ice. An aliquot of 100µl was counted using a γ-counter (1470 Wizard Automatic Gamma Counter from Perkin Elmer (UK)) and the remaining was centrifuged at 8050×g for 3 min at 4°C to obtain plasma. A 50µl aliquot of plasma was subsequently counted using the γ-counter. An aliquot of the remaining plasma from each sample was mixed with 5% of perchloric acid (PCA) (v/v) to precipitate proteins and centrifuged at 8050×g for 5 min at 4°C. The supernatant was counted on the γ-counter to measure the amount of free [^{11}C]leucine in plasma. Free and protein-incorporated [^{11}C]leucine levels were calculated from the difference between total [^{11}C]leucine in plasma and the supernatant from the PCA precipitation (free [^{11}C]leucine). A last aliquot of plasma was stored at -80°C until sent to the Alta Bioscience Ltd (Redditch, UK, https://altabioscience.com) for the analysis of LNAA and unlabelled leucine in plasma.

Population Image-derived IF (IDIF)

Individual AIF were obtained in Wistar (n=6) and 12 (n=3) and 18 months old (n=6) Fisher-344 rats. AIF from the F344 rats were temporally aligned and pooled together to generate a population-based IF (PBIF, Figure S1). In the longitudinal study, in which most rats did not have an individual AIF, a heart left-ventricle (LV) ROI was manually selected based on the automatic segmentation performed in BrainVisa 4.1.1[13, 14] to determine a whole-blood time activity curve (TAC). The 180-240 seconds timeframe was selected because the SUV values from the averaged

LV TAC were identical to the averaged AIF measured by γ-counting and Twilite. The ratio between the population-based IF and each individual LV TAC for animals without AIF was calculated and use to scale the population-based IF for blood [^{11}C]leucine SUV (Figures S1 & S2).
